# 

**Published:** 1874-01

**Authors:** 


### CONTENTS:

ORIGINAL COMMUNICATIONS:

ARTICLE I. On a Marsh Plant from the Mississippi River Ague Bottoms, supposed to be kindred to the Gemiasma of Salisbury; with a Consideration of its Genetic Relations to Malarial Diseases. Read before the Chicago Society of Physicians and Surgeons, Nov. 10, 1873. By JOHN BARTLETT, M.D., Chicago .......... 1

SELECTIONS:

Bite of the Diamond Rattlesnake (Crotalus Adamanteus). By A. Mitchell, M.D .......... 42

Facts and Theories about the Recent Outbreak of Asiatic Cholera. By J. C. Peters, M.D .......... 44

A Case of Rheumatic Fever treated with a Cold Bath; Death occurring immediately on leaving the Bath. By Sidney Ringer, M.D .......... 49

Recovery from Bite of a Rattlesnake. By W. F. C. Beattie, M.D .......... 51

EDITORS' BOOK TABLE .......... 52

EDITORIAL .......... 61

OBITUARY .......... 64

